# Severe refractory autoimmune hemolytic anemia with both warm and cold autoantibodies that responded completely to a single cycle of rituximab: a case report

**DOI:** 10.1186/1752-1947-5-156

**Published:** 2011-04-19

**Authors:** Shilpi Gupta, Anita Szerszen, Fadi Nakhl, Seema Varma, Aaron Gottesman, Frank Forte, Meekoo Dhar

**Affiliations:** 1 Department of Medicine (Hematology and Medical Oncology), Staten Island University Hospital, 256 C Mason Avenue, Staten Island, NY 10305, USA; 2 Department of Medicine (Geriatrics), Staten Island University Hospital, 475 Seaview Avenue, Staten Island, NY 10305, USA; 3 Department of Medicine (Hospitalist Medicine), Staten Island University Hospital, 475 Seaview Avenue, Staten Island, NY 10305, USA

## Abstract

**Introduction:**

Mixed warm and cold autoimmune hemolytic anemia runs a chronic course with severe intermittent exacerbations. Therapeutic options for the treatment of hemolysis associated with autoimmune hemolytic anemia are limited. There have been only two reported cases of the effective use of rituximab in the treatment of patients with mixed autoimmune hemolytic anemia. We report a case of severe mixed autoimmune hemolytic anemia that did not respond to steroids and responded to four weekly doses of rituximab (one cycle).

**Case presentation:**

A 62-year-old Caucasian man presented with dyspnea, jaundice and splenomegaly. His blood work revealed severe anemia (hemoglobin, 4.9 g/dL) with biochemical evidence of hemolysis. Exposure to cold led to worsening of the patient's hemolysis and hemoglobinuria. A direct antiglobulin test was positive for immunoglobulin G and complement C3d, and cold agglutinins of immunoglobulin M type were detected. A bone marrow biopsy revealed erythroid hyperplasia. A positron emission tomographic scan showed no sites of pathologic uptake. There was no other evidence of a lymphoid or myeloid disorder. Initial therapy consisted of avoidance of cold, intravenous methylprednisolone and a trial of plasmapheresis. However, there was no clinically significant response, and the patient continued to be transfusion-dependent. He was then started on 375 mg/m^2^/week intravenous rituximab therapy. After two treatments, his hemoglobin stabilized and the transfusion requirement diminished. Rituximab was continued for a total of four weeks and led to the complete resolution of his hemolytic anemia and associated symptoms. At the patient's last visit, about two years after the initial rituximab treatment, he continued to be in complete remission.

**Conclusion:**

To the best of our knowledge, this is the first reported case of mixed-type autoimmune hemolytic anemia that did not respond to steroid therapy but responded completely to only one cycle of rituximab. The previous two reports of rituximab use in mixed autoimmune hemolytic anemia described an initial brief response to steroids and the use of rituximab at the time of relapse. In both of these case reports, the response to one cycle of rituximab was short-lived and a second cycle of rituximab was required. Our case report demonstrates that severe hemolysis associated with mixed autoimmune hemolytic anemia can be unresponsive to steroid therapy and that a single cycle of rituximab may lead to prompt and durable complete remission.

## Introduction

Autoimmune hemolytic anemia (AIHA) is one of the most common causes of acquired hemolytic anemia. The cause of AIHA remains idiopathic in 50% of the cases [1]. The clinical presentation of AIHA depends on the subclass type: warm agglutinin, cold agglutinin and mixed disorder, as well as the thermal range activity of the causative autoantibody.

Mixed warm and cold AIHA runs a chronic course with severe intermittent exacerbations. Therapeutic options for the treatment of hemolysis associated with mixed AIHA are limited.

Therapeutic options for patients with AIHA include treatment of the underlying etiology, such as a lymphoproliferative disorder if diagnosed, or the use of cytotoxic agents such as cyclophosphamide, cyclosporine, chlorambucil or corticosteroids. Additionally, plasmapheresis can be used for the removal of causative antibodies and to slow down the rate of hemolysis. Splenectomy has been employed in patients with warm autoimmune hemolytic disease to slow down the hemolysis. Recently, reports of the use of rituximab for initial and recurrent cases of AIHA have shown an objective response, with more than 50% of patients experiencing complete remission [2]. We have utilized this treatment with promising results.

## Case presentation

A 62-year-old Caucasian man with a history of chronic alcohol abuse presented to the emergency department with complaints of shortness of breath and confusion of three days' duration. The patient's vital signs were stable except for sinus tachycardia of 110 beats/min. The patient was confused, lethargic and pale. His physical examination was remarkable for scleral icterus, shifting dullness, hepatosplenomegaly and bilateral lower-extremity pitting edema. There was no significant peripheral lymphadenopathy, and there was no evidence of hypertension.

The complete blood count revealed anemia with a hemoglobin level of 4.5 g/dL, a reticulocyte count of 25.82% and normal white cell and platelet counts. Hemolysis was confirmed by elevated lactate dehydrogenase (LDH) of 447 U/L and low haptoglobin of 9.18 mg/dL. Red blood cell agglutination, polychromasia, target cells and spherocytes were seen on a peripheral smear. The direct Coombs test was positive for complement C3d and immunoglobulin G (IgG) antibody, which were identified as anti-I cold agglutinins at 4°C.

The diagnosis of renal insufficiency was made on the basis of the patient's glomerular filtration rate of 40.84 mL/min/1.73 m^2 ^(creatinine level of 2 mg/dL). His liver function tests were significant for an elevated total bilirubin level of 5.3 mg/dL, a direct bilirubin level of 1.8 mg/dL and an ammonia level of 136 μM/L. His albumin and total protein levels were 3.1 g/dL and 7 g, respectively. The patient's hepatitis profile was negative, and no cryoglobulinemia was observed.

Although our patient did not have gastrointestinal bleeding or hematuria, his urine was reported to be dark on several occasions, which was precipitated by exposure to cold. Additionally, marked drops in hemoglobin and haptoglobin levels were noted after exposure to cold. Proteinuria on urine analysis prompted a 24-hour urine collection, which was significant for nephrotic range proteinuria of 15 g/day. Serum whole complement activity (CH50) level was reduced with normal complement C3 and C4 levels. No monoclonal spike was observed in serum or urine electrophoresis.

*Mycoplasma pneumoniae *infection, infectious mononucleosis, systemic lupus erythematosus and human immunodeficiency virus were ruled out. Computed tomography (CT) of the chest, abdomen and pelvis was remarkable for hepatosplenomegaly. Subsequently, the patient underwent a bone marrow biopsy that showed a hypercellular marrow with erythroid hyperplasia, but no evidence of dysplasia or lymphoma. A liver biopsy revealed stage IV fibrosis with no evidence of malignancy. This finding was thought to be secondary to the history of alcohol abuse.

During the hospital course, the patient underwent transfusion with several units of incompletely matched packed red blood cells through a warmer and was started on intravenous methylprednisolone therapy. In spite of corticosteroid therapy, the patient's hemoglobin did not improve, and he continued to require blood transfusions almost daily. Consequently, a daily regimen of plasmapheresis was initiated.

**Figure 1:**
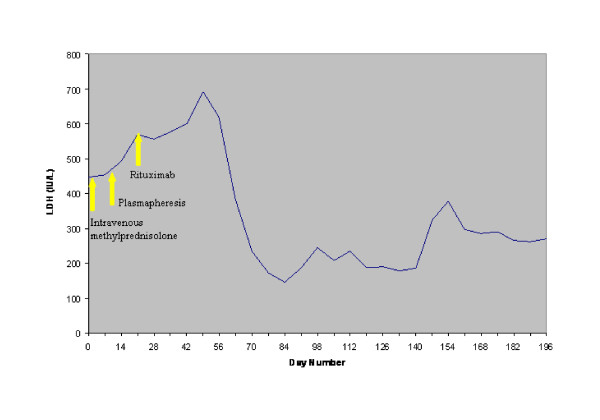
**Effect of treatment on serum lactate dehydrogenase**.

**Figure 2:**
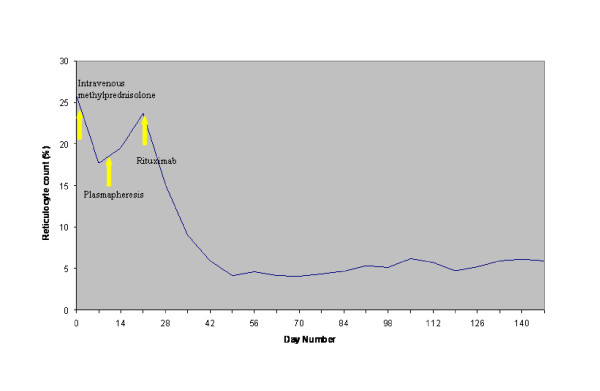
**Effect of treatment on reticulocyte count**.

**Figure 3:**
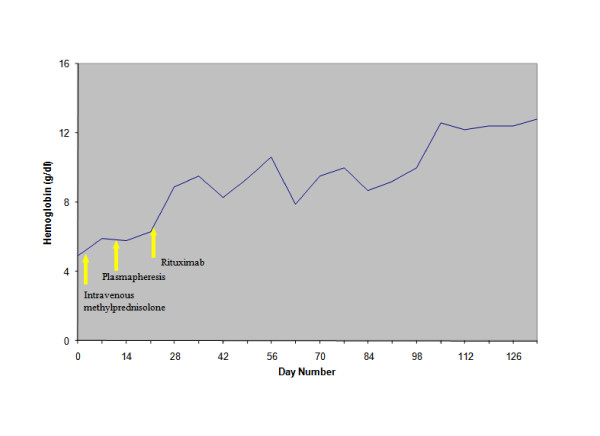
**Effect of treatment on hemoglobin levels**.

Partial resolution of the hemolytic process was observed while the patient was treated with daily plasmapheresis with 5% albumin, at a volume of 3L to 4L. A total of seven daily plasmapheresis treatments were performed, which resulted in a gradual decrease of the patient's LDH and bilirubin and a rise in his level of haptoglobin. However, the patient still required almost daily blood transfusions. On the basis of earlier reports indicating an anecdotal benefit of rituximab treatment for immune cytopenias, plasmapheresis was discontinued and our patient was placed on rituximab therapy at a dose of 375 mg/m^2 ^every week. A total of four doses were administered over a period of four weeks. Although an initial increase in LDH level after the initiation of rituximab treatment was noted, there was no evidence of worsening hemolysis. After the first two courses of rituximab therapy, the patient showed a marked clinical improvement. His hemoglobin level stabilized (Figure 1, Figure 2 and Figure 3), and he no longer required blood transfusions.

Subsequently, he was discharged to home with continued follow-up as an outpatient. He was also started on β-blockers and calcium channel blockers for high blood pressure at the time of discharge. During outpatient follow-up, the patient's proteinuria level decreased from 15 g per 24 hours to 5.5 g per 24 hours. A renal biopsy was performed and demonstrated nodular glomerulosclerosis with no evidence of immune complex deposition. At his two-year follow-up examination after the initial rituximab therapy, the patient continued to be transfusion-independent and his last hemoglobin level was 12.5 g/dL. Another renal biopsy was performed and demonstrated nodular mesangial widening with increased matrix and tubular basement membrane thickening without deposits visualized on light microscopy. On the basis of the findings of light and electron microscopy and immunopathology, a diagnosis of nodular glomerulosclerosis with no evidence of immune complex deposition was made. These changes can be seen in patients with diabetes or even in healthy individuals. At his two-year follow-up examination after the initial rituximab therapy, the patient continued to be transfusion-independent and his last hemoglobin level reading was 12.5 g/dL. His hospital course was complicated by the development of steroid-induced diabetes mellitus, warranting insulin use during his hospitalization. His diabetes resolved after the discontinuation of steroids.

## Discussion

On the basis of the clinical presentation and laboratory analysis, our patient was diagnosed with mixed AIHA. Concomitant features of liver dysfunction and nephrotic syndrome secondary to nodular glomerulosclerosis were also seen. Reticuloendothelial involvement led to an extensive workup that ruled out an infectious or lymphoproliferative etiology of this clinical presentation.

Rituximab is a anti-CD20 monoclonal antibody that has been used in the management of patients with cold agglutinin disease with severe hemolysis not responding to treatment with conventional therapy. In an uncontrolled prospective study, 14 of 27 patients with cold agglutinin disease, 15 of whom had been previously treated, responded to a single course of rituximab, and six of 10 responded to retreatment with rituximab and interferon [2]. In another study, rituximab was used to treat immune cytopenia in adults, and 40% of the patients with AIHA showed complete remission [3]. The use of rituximab in a small prospective study of eight patients with nephrotic syndrome due to idiopathic membranous nephropathy led to sustained disease remission [4].

In view of the limited response to conventional therapy with corticosteroids and plasmapheresis, rituximab therapy was initiated, which led to rapid improvement in our patient and marked improvement in the hemolytic process. His hemoglobin level stabilized, and he did not require blood transfusion after rituximab therapy. His proteinuria was reduced by more than 50%, and his edema almost completely resolved. The nodular glomerulosclerosis noted on the patient's renal biopsy may have been idiopathic or secondary to diabetic nephropathy, immunotactoid glomerulonephritis, fibrillary glomerulonephritis, cryoglobulinemic glomerulonephritis, amyloidosis, light-chain deposition disease or heavy-chain deposition disease. The cause is unclear; however, the autoimmune disorder that caused the AIHA might also have been a precipitating factor for the renal findings.

Rituximab has been shown to be effective in the treatment of viral infection-associated nephropathy in conjunction with antiviral therapy. Ohsawa *et al*. [5] reported the case of a patient with cryoglobulinemia and hepatitis C virus infection. Their patient had warm antibody-mediated AIHA with immune complex nephropathy.

To our knowledge, our case is the first reported presentation of mixed AIHA that did not respond to steroids but showed a complete and sustained response to rituximab. Two previous reports of rituximab use in mixed AIHA described an initial brief response to steroids. Rituximab was begun at the time of relapse. In both cases, the response to four weekly injections of rituximab was short-lived and required a second cycle [6,7].

## Conclusion

This is the first reported case of a patient with mixed-type AIHA who did not respond to steroid therapy but showed a complete response to only one cycle of rituximab. Refractory AIHA is a difficult condition to manage, and novel therapeutic agents such as rituximab merit further investigation in this setting.

## Consent

Written informed consent was obtained from the patient for publication of this case report and any accompanying images. A copy of the written consent is available for review by the Editor-in-Chief of this journal.

## Conflict of interests

The authors declare that they have no competing interests.

## Authors' contributions

SG wrote the manuscript. AS performed the literature search and helped with writing the manuscript. FN performed the literature search and all procedures required for the patient. SV constructed all the figures in the manuscript. AG performed the literature search. FF was a major contributor in the writing of the manuscript. MD was the treating physician of the patient. All authors read and approved the final manuscript.
